# Eicosapentaenoic and docosahexaenoic acid supplementation during early gestation modified relative abundance on placenta and fetal liver tissue mRNA and concentration pattern of fatty acids in fetal liver and fetal central nervous system of sheep

**DOI:** 10.1371/journal.pone.0235217

**Published:** 2020-06-23

**Authors:** José Alejandro Roque-Jimenez, Mario Francisco Oviedo-Ojeda, Megan Whalin, Héctor Aaron Lee-Rangel, Alejandro Enrique Relling

**Affiliations:** 1 Universidad Autónoma de San Luis Potosí, Facultad de Agronomía, Soledad de Graciano Sánchez, San Luis Potosí, México; 2 Department of Animal Science, The Ohio State University, Ohio Agricultural Research and Development Center (OARDC), Wooster, OH, United States of America; Universidade Federal de Viçosa, BRAZIL

## Abstract

In sheep, polyunsaturated fatty acid (PUFA) supplementations in late gestation increases the growth of offspring; however, there is a lack of evidence on the effect of PUFA supplementation during early gestation. Thus, the objective of this study was to evaluate the effect of dietary supplementation of eicosapentaenoic acid (EPA) and docosahexaenoic acid (DHA) in early gestation pregnant ewes on fatty acid concentration of fetal liver (FL) and fetal central nervous system (FCNS), and relative abundance of the mRNA for genes associated with transport and metabolism of fatty acids in FL and placenta. A total of 12 ewes, block for stage of gestation were fed a diet containing 1.6% (dry matter basis) monounsaturated fatty acids (MUFA) or EPA+DHA during the first 45 days of gestation. A cesarean section was conducted on day 45 of gestation to collect placenta (caruncle and cotyledon), FL, and FCNS. Relative abundance of mRNA in FL and FCNS and fatty acid concentration were analyzed using a 2x2 factorial arrangement of treatments considering fatty acid supplementation and tissue as the main factors. Concentrations of C18:1 isomers increase (*P < 0*.*05*) in FL and FCNS with MUFA supplementation; the FL and FCNS had a greater concentration of C20:3(n-6), C20:3(n-3), C22:1, C22:5 and C22:6 (*P < 0*.*05*) with EPA+DHA supplementation. In FL, the relative abundance of *LPL* mRNA was greater *(P = 0*.*02*) as a result of MUFA supplementation. In placenta, there was a FA x tissue interaction for relative abundance of *DNMT3b* and *FFAR-4* mRNA (*P < 0*.*05*). Fetus from MUFA-supplemented dams had a greater relative abundance of *FABP-4* mRNA (*P < 0*.*05*). Results indicate supplementation with EPA+DHA during early gestation increases the total EPA and DHA in FL. For the placenta, EPA+DHA supplementation led to an increase in the relative abundance of lipid mRNA for transport genes.

## Introduction

Omega-3 polyunsaturated fatty acids (n-3 PUFA) are essential nutrients during gestation, as well as fetal development and as adults. Considerable research has focused on EPA (C20:5) and DHA (C22:6) supplementation during gestation in mammals [1]. Both fatty acids (FA) have beneficial effects on the support of normal growth and development of various tissues during fetal development. Some of the effects of DHA and EPA are important for fetal brain development, specific components of the neural system, retinal maturation, and neonatal behavior [2]. The placenta regulates nutrient transfer from the dam to the fetus [3]. Ewe placentas have placentomes, of which the caruncles and cotyledons are the primary functional tissues, and its physiological function is the exchange of nutrients, such as FA, between dam and fetus [4, 5]. The mechanisms underlying placental uptake, metabolism, and transfer of EPA and DHA are complex and not fully understood.

Omega-3 PUFA in maternal circulation are bound to fatty acid transport proteins (*FATP*), plasma membrane fatty acids binding protein (*FABPpm*), and fatty acid translocase (*FAT/CD36*) [6]. Furthermore, the proteins encoded by the *FABPpm* and *FAT/CD36* genes regulate the transport of FA through the placenta [7]. Results of studies in dairy cows indicate that free FA receptors (*FFAR*) are activated by FA, regulating lipid metabolism and placenta functions [8, 9]. Dietary DHA increases the abundance of DNA methyltransferase (*DNMT*) proteins in rodent placentas and FLs [10, 11]. The increase in the protein encoded by the *DNMT* gene has been associated with changes in the pattern of FA concentrations in the fetal liver (FL) [12]. Even though there is knowledge of FA transport and function in the placenta, there is lack of knowledge about the association of maternal supplementation in early gestation with EPA and DHA supplementation and changes in abundance of *FATP*, *FFAR* and *DNMT* mRNA transcripts on caruncle and cotyledon. Through different pathways, fatty acids regulate the expression of a range of genes involved in lipid and lipoprotein metabolism within the liver [13]. Much of the existing evidence relates specifically to adult mice and fish; however, there is much less understanding of the potential functions of EPA and DHA on the FL. It is not clear if EPA and DHA supplementation during early gestation have the capacity to alter fetal pathways involved in lipid and lipoprotein metabolism of the FL and FCNS. Supplementation with EPA and DHA during pregnancy led to modification of the concentration of EPA and DHA of the FL and FCNS [14]. In different species the supplementation with EPA and/or DHA, however, results differing outcomes for FA concentration or relative abundance of relevant mRNA transcripts in the liver [15].

The hypothesis of the present study is that supplementation with EPA and DHA during the first third of gestation in pregnant ewes increases the concentration of EPA and DHA in FL and central nervous system; and changes the relative abundance of mRNA transcripts for genes associated with fatty acid transport and metabolism in the FL and placenta. The objectives of the current study were to determine the effect of diet supplementation with EPA and DHA to the dam in early gestation on changes in the concentration of EPA and DHA of the FL and central nervous system; and on relative abundances of mRNA transcripts of genes associated with transport and metabolism of FA, in FL and placenta.

## Materials and methods

### Animals, experimental design and treatments

All animal procedures were approved by the Agricultural Animal Care and Use Committee of The Ohio State University (IACUC # 2016A00000013). Using a randomized complete block design there was assignment to two treatments groups of 12 gestating ewes (6/treatment) blocked by day of gestation. Ewes were housed with a ram and once a standing estrus was confirmed, ewes were removed from the group pen and randomly located in smaller pens. Ewes were housed in pens with two ewes per pen (six pens); and pen was considered the experimental unit. The treatments, randomly assigned to the pens, were: 1) Ca salts of a palmitic fatty acid distillate (MUFA; EnerGII, Virtus Nutrition LLC, Corcoran, CA), and 2) Ca Salts containing EPA and DHA (EPA+DHA; StrataG113, Virtus Nutrition LLC, Corcoran, CA). The diet was a mixed ration containing 50% corn silage, 32.175% soy hulls, 16.09% distiller grains, 1.61% of Ca salts and 0.125% of mineral and vitamin mix (**Table 1**). The diet was formulated to meet or exceed the nutrient requirements [16] for ewes during early gestation. The dose of fatty acid supplementation was based on previous research in pregnant ewes [17, 18] where supplementation at similar doses had effects on lamb growth and abundances of relevant mRNA transcripts in the adipose tissue of ewes [18].

**Table 1:** Formulation and chemical composition (% DM basis) of the basal diet fed to pregnant ewes during the first 45 days of gestation.

	Treatment	
Item, g/Kg DM	MUFA^1^	EPA+DHA^2^
**Ingredient**		
** Corn Silage**	50.00	50.00
** DDGS**^3^	16.09	16.09
** Soy Hulls**	32.18	32.18
** MUFA Ca Salts (EnerGII, Virtus Nutrition LLC)**	1.61	-
** PUFA Ca Salts (EPA+DHA; StrataG113, Virtus Nutrition LLC)**	-	1.61
** Pre-mix Minerals and Vitamins**	0.13	0.13
**Chemical Composition**^4^		
** Neutral detergent fiber**	43.98	43.41
** Acid detergent fiber**	28.12	26.38
** CP**	13.21	13.38
** Ca**	0.43	0.45
** P**	0.27	0.28
** Ash**	4.86	5.07
** Ether extract**	4.16	3.77

^1^Ca salts of a palmitic fatty acid distillate (MUFA; EnerGII, Virtus Nutrition LLC, Corcoran, CA)

^2^Ca Salts of containing EPA and DHA (EPA+DHA; StrataG113, Virtus Nutrition LLC, Corcoran, CA)

^3^Distillers dried grains with solubles; Dakota Gold (Marion, OH)

^4^CP (Crude Protein), Ca (Calcium), P (Phosphate)

### Sampling

Feed samples were collected weekly, pooled and analyzed according to AOAC [19] for dry matter (DM, method number 981.10), crude protein (CP, method number 967.03) and NDF and ADF using the procedures previously reported by Van Soest et al. [20] with a heat-stable amylase included in the analysis. Total fatty acids composition of Ca salts was determined using the methods described by Weiss and Wyatt [21] (**Table 2**).

**Table 2:** Fatty acid profile (% of total FA) relative to source of Ca salts from a palmitic fatty acid distillate (MUFA) or Ca Salts containing EPA and DHA (EPA+DHA) supplemented in the feed of pregnant ewes during the first 45 days of gestation.

	Supplement^1^^,^^2^	
Fatty acid	MUFA	EPA+DHA
**C8:00.110.00C10:00.020.00C12:0**	0.62	0.12
**C14:0**	1.17	5.99
**C16:0**	45.87	22.01
**C16:1**	0.20	7.40
**C18:0**	5.14	7.47
**C18:1 c9**	36.27	17.46
**C18:1 other**	1.10	4.51
**C18:2**	8.03	2.69
**C20:0**	0.37	0.34
**C20:1**	0.09	0.84
**C18:3**	0.20	0.94
**C22:0**	0.00	0.35
**C22:1**	0.00	1.38
**C20:3 n-3**	0.00	0.51
**C20:4**	0.00	0.00
**C20:5**	0.13	9.19
**C22:6**	0.00	7.00
**Other**	0.80	12.15

^1^MUFA, EnerGII as a source of palmitic and oleic acid (Virtus Nutrition LLC, Corcoran, CA); EPA+DHA, StrataG113 as a source of eicosapentaenoic acid and docosahexaenoic acids (Virtus Nutrition LLC).

^2^Fatty acid profiles evaluated using the methods of Weiss and Wyatt (41).

After 45 days of supplementation with MUFA or EPA+DHA, a caesarian section was conducted to collect placenta and fetal samples. From the 12 ewes, 11 were single-gestation, and one, on the MUFA treatment, was a twin-gestation. From the twin gestation ewe only one fetus was randomly sampled. Feed was withheld for 12 hours prior to surgery. The ewes were sedated with xylazine 0.2 mg/kg IM. Once recumbent, ewes were intubated and there was anesthesia indication using Isoflurane 3% to 5% and anesthesia maintenance throughout the procedure. Ewes were placed in dorsal recumbency and the ventral area was clipped to remove wool and scrubbed three times with betadine and alcohol. A 20 cm incision was made from front of the udder toward the umbilicus extending through *linea alba* and peritoneum. The uterus and ovaries were exteriorized. The ovarian pedicle was ligated twice and transected with type 0 surgical gut. The uterine body was clamped, and suture ligation using type 0 surgical gut, twice, cranial to the cervix. The peritoneum and *linea alba* were closed using a type 0 gut with a continuous suturing procedure. The skin was closed using a type 1 gut by performing a cruciate procedure. The tissues were removed using sterile scalpel and forceps, placed into cryogenic vials (Thermo Fisher Scientific, Waltham, MA), and snap frozen using liquid nitrogen and stored at -80°C until analyses were conducted. The caruncle and cotyledon were separated using procedures that were described [22]. For the FCNS and FL the entire brain and liver were collected.

#### Fetal liver and fetal central nervous system fatty acid analysis

For fatty acid composition of FL and FCNS determinations, there was use of 100 to 150 mg of the FL and FCNS samples that were placed in a 16 x 125 mm screw cap culture tube. The method used for FA extraction and methylation of these tissues, including the internal standard and the reagents used in the different steps of the procedures were previously described by O’Fallon et al. [23]. The extracted and methylated samples were stored in gas **chromatography (**GC) cap vials and stored at -20°C until analysis. All fatty acid methyl esters were separated using gas liquid chromatography utilizing a CP-SIL88 capillary column (100-m x 0.25-mm x 0.2-μm film thickness; Varian Inc., Palo Alto, CA).

#### Fetal liver, caruncle, and cotyledon mRNA abundances

The FL, caruncle, and cotyledon tissues were homogenized and isolated using the TRI Reagent^TM^. The extraction of RNA was performed using a commercial kit with centrifugations and DNAse digestion (R2070 Direct-zol^TM^ RNA miniprep Plus, Zymo Research, USA) being conducted using the manufacturer’s protocol. Extracted RNA from all samples was quantified using UV spectroscopy (Nanodrop Technologies) and qualitatively assessed using a BioAnalyzer.

Relative abundances of mRNA for the genes of interest was determined using a Nanostring nCounter XT Assay (Nanostring Technologies, WA) for 28 genes selected based on the functions on fatty acid transport proteins, intracellular fatty acid binding proteins, nuclear receptors, transfer enzymes of methyls group, hormone receptors and housekeeping. Those genes were: *Diacylglycerol O-acyltransferase 1 (DGAT1); Diacylglycerol O-acyltransferase 2 (DGAT2); DNA methyltransferase 1 (DNMT1); DNA methyltransferase 2 (DNMT2); DNA methyltransferase 3a (DNMT3a); DNA methyltransferase 3b (DNMT3b); Fatty acid elongase 2 (ELOVL2); Fatty acid elongase 5 (ELOVL5); Fatty acid binding protein 1 (FABP-1); Fatty acid binding protein 4 (FABP-4); Fatty acid binding protein 5 (FABP-5); Fatty acid desaturase 1 (FADS1); Fatty acid translocase (FAT/CD36); Fatty acid transport protein 1 (FATP-1); Fatty acid transport protein 4 (FATP-4); Free fatty acid receptors 1 (FFAR-1); Free fatty acid receptors 4 (FFAR-4); Glucose transporter 1 (GLUT1); Insulin like growth factor 2 (IGF-2)(AR-4*,*6*, *sferasesedoncryogenic vials and stored at -80°C until analysis*. *Endothelial lipase precursor (LIPG); Lipoprotein lipase (LPL); Peroxisome proliferator activated*: *alpha*, *Betha*, *Gamma (PPAR α*, *β*, *γ); Stearoyl CoA desaturase (SCD); Sterol regulatory element binding protein 1 (SREBP-1)* (Table 3). The abundance of specific target molecules was then quantified using the nCounter digital analyzer. Individual fluorescent barcodes and target molecules present in each sample were recorded with a charge-coupled device camera by performing a high-density scan (325 fields of view). Images were processed internally into a digital format and exported as Reporter Code Count (RCC) files [17]. The nSolver Analysis Software 3.0 (Nanostring Technologies, Seattle, WA) was used to analyze nCounter data. Briefly, RCC files were uploaded and data were normalized to the geometric mean of the housekeeping target genes: *Apoliprotein B* (nor*ApoB); Tata box binding protein (TBP); Ciclophilin B (CypyB); Phosphoglycerate kinase (PGK1); Polymerase I polypeptide B (POLR1B)*. The effect of the treatment on the abundance of mRNA housekeeping genes was evaluated, and there were no treatment effects on abundances of any of the five mRNA transcripts of genes of the tissues. The five housekeeping target genes, therefore, were used to normalize the data (Table 3).

**Table 3:** Genes names and Gen Bank accession number used to determine the relative abundance of mRNA transcripts.

Gene name ^a^	Accession number
*DGAT1*	NM_001110164.1:555
*DGAT2*	XM_012096078.2:1459
*DNMT1*	NM_001009473.1:1307
*DNMT2*	AY244708.1:876
*DNMT3a*	XM_012166008.2:2152
*DNMT3b*	XM_012189044.2:1336
*ELOVL2*	XM_012101293.2:1786
*ELOVL5*	XM_012100862.2:1984
*FABP-1*	XM_004005898.3:244
*FABP-4*	NM_001114667.1:195
*FABP-5*	NM_001145180.1:256
*FADS-1*	XM_012101996.2:1086
*FAT/CD36*	XM_012176587.2
*FATP-1*	XM_015095580.1
*FATP-4*	XM_015094163.1
*FFAR1*	XM_015100194.1:227
*FFAR4*	XM_012102571.2:1086
*GLUT1*	XM_015091913.1
*IGF-1*	NM_001009774.3:242
*IGF-2*	NM_001009311.1:420
*LIPG*	XM_012103679.2:1337
*LPL*	NM_001009394.1:724
*Leptin*	XM_004008038.3:2018
*PPAR β*	XM_004018768.3:474
*PPAR α*	XM_012175774.2:1083
*PPAR γ*	NM_001100921.1:640
*SCD*	NM_001009254.1:1132
*SREBP-1*	XM_015098336.1:3946
*ApoB* *	XM_012175938.1:3919
*TBP* *	XM_015097549.1
*CypyB* *	XM_004010536.3
*PGK1* *	NM_001142516.1:643
*POLR1B* *	XM_004005912.3:2371

^a^ DGAT1 = *Diacylglycerol O-acyltransferase 1*; DGAT2 = *Diacylglycerol O-acyltransferase 2*; DNMT1 = *DNA methyltransferase 1*; DNMT2 = *DNA methyltransferase 2*; DNMT3a = *DNA methyltransferase 3a*; DNMT3b = *DNA methyltransferase 3b;* ELOVL2 = *Fatty acid elongase 2*; ELOVL5 = *Fatty acid elongase 5*; FABP-1 = *Fatty acid binding protein 1*; FABP-4 = *Fatty acid binding protein 4*; FABP-5 = *Fatty acid binding protein 5*; FADS1 = *Fatty acid desaturase 1*; FAT/CD36 = *Fatty acid translocase*; FATP-1 = *Fatty acid transport protein 1*; FATP-4 = *Fatty acid transport protein 4*; FFAR-1 = *Free fatty acid receptors 1*; FFAR-4 = *Free fatty acid receptors 4*; GLUT-1 = *Glucose transporter 1*; IGF-1 = *Insulin like growth factor 1*; IGF-2 = *Insulin like growth factor 2*; LIPG = *Endothelial lipase precursor*, LPL = *Lipoprotein lipase*; PPAR β = *Peroxisome proliferator activated betha*; PPAR α = *Peroxisome proliferator activated alpha;* PPAR γ = *Peroxisome proliferator activated gamma*; SCD = *Stearoyl CoA desaturase*; SREBP-1 = *Sterol regulatory element binding protein 1*; *Apoliprotein B (norApoB); Tata box binding protein (TBP); Ciclophilin B (CypyB); Phosphoglycerate kinase (PGK1); Polymerase I polypeptide B (POLR1B)*^.^

*genes selected for normalized all data to the geometric mean of the housekeeping target genes.

### Statistical analysis

Fatty acid concentration from FL and FCNS were analyzed using the MIXED procedure of SAS (9.4) for a randomized complete block design with repeated measurements using a 2 x 2 factorial arrangement of treatments. The statistical model used was:

$$Y_{ijk}=\mu +Fi+T_{j}+FT_{ij}+B_{k}+e_{ijk},\;where:$$

T_i_ = the fixed effect of type of FA supplemented (MUFA compared with EPA+DHA),

F_j_ = the fixed effect of the tissue (FL compared with FCNS),

FT_ij_ = the fixed effect of the interaction of FA supplementation and tissue,

B_k_ = the random effect of block, and

e_ijk_ = random error.

The repeated measurement was added into the model to remove the lack of independence between the FA concentration data of the FL and FCNS of the same fetus. For the FL relative abundance of mRNA transcripts, a similar model of the FA was used using only one factor (FA supplemented) without the effect of tissue, its interaction, and the repeated measurements. For relative abundance of placenta mRNA transcripts, the same model of FA concentration was used, but the second factor considered was the two sides of the placenta (caruncle compared with cotyledon).

## Results

### Fetal liver and fetal central nervous system fatty acid

The concentration of C22:1 increased in the FL from MUFA supplemented dams (treatment by tissue interaction *P =* 0.01) compared with the FCNS from MUFA supplemented dams and tissues from fetus from EPA+DHA supplemented dams (Table 4). For C20:5 the concentration increased in the FL and the FCNS from EPA+DHA supplemented dams (treatment by tissue interaction *P =* 0.01) compared with the tissues from fetus from MUFA supplemented dams (Table 4). The concentration of C24:0 increased in the FL from EPA+DHA supplemented dams (treatment by tissue interaction *P =* 0.01) compared with the FCNS from EPA+DHA supplemented dams and tissues from fetus from MUFA supplemented dams (Table 4). The concentration of C18:3 was greater in the FCNS of MUFA-supplemented dams (treatment by tissue interaction *P =* 0.01) compared with the FL of MUFA-supplemented dams and tissues from the fetus of EPA+DHA-supplemented dams (Table 4). The concentration of C20:3 n-3 was greater in the FL of MUFA supplemented dams (treatment by tissue interaction *P =* 0.01) compared with the FCNS of MUFA-supplemented dams and tissues from fetus of EPA+DHA-supplemented dams (Table 4). The total PUFA concentration tended to decrease (*P =* 0.08) in the FCNS of MUFA-supplemented dams compared with the FL of MUFA-supplemented dams and tissues from fetus of EPA+DHA supplemented dams (Table 4).

**Table 4:** Effects of supplementation with source of Ca Salts of a palmitic fatty acid distillate (MUFA) (n = 6) or Ca Salts containing EPA and DHA (EPA+DHA) (n = 6) on the fatty acids profile of fetal liver and central nervous system in lambs of ewes supplemented during the first 45 days of gestation.

Item	Treatment ^a^				SEM	*P-value*		
	MUFA		EPA+DHA					
	FL	FCNS	FL	FCNS		Treatment	Tissue	Tissue x treatment
**C14:0**	1.12	2.27	1.17	2.10	0.13	0.70	< 0.01	0.36
**C15:0**	0.27	0.06	0.30	0.08	0.04	0.55	0.01	0.75
**C16:0 iso**	0.43	1.80	0.38	1.44	0.11	0.09	< 0.01	0.21
**C16:0**	18.58	20.99	18.82	19.87	0.70	0.51	0.04	0.38
**C17:0 iso**	0.17	0.30	0.05	0.33	0.14	0.76	0.18	0.61
**C16:0 & C17:0 ante**	1.35	1.36	1.46	1.39	0.06	0.38	0.59	0.50
**C17:0**	0.56	0.02	0.42	0.00	0.07	0.28	0.01	0.46
**C17:1**	0.33	0.63	0.32	0.58	0.05	0.50	0.01	0.79
**C18:0**	14.69	10.39	13.02	10.32	0.46	0.12	< 0.01	0.07
**C18:1 t6,8**	0.24	0.26	0.12	0.08	0.04	0.01	0.80	0.48
**C18:1 t12**	0.67	0.55	0.55	0.25	0.07	0.01	0.01	0.22
**C18:1 c13**	0.00	0.00	0.00	0.05	0.02	0.34	0.34	0.34
**C18:1 c9**	11.51	10.55	11.19	10.28	0.54	0.63	0.07	0.95
**C18:1 c11**	3.70	2.91	3.44	2.78	0.16	0.38	< 0.01	0.48
**C18:1 c16**	0.07	0.00	0.04	0.00	0.02	0.65	0.03	0.65
**C18:1 c15**	0.00	0.00	0.11	0.00	0.02	0.06	0.06	0.06
**C19:0**	22.56	34.28	25.62	34.55	2.44	0.53	0.01	0.55
**C18:2 c9 t12**	1.07	0.26	1.30	0.34	0.07	0.07	< 0.01	0.32
**C20:0**	0.17	0.06	0.24	0.04	0.03	0.38	0.01	0.37
**C18:3**	0.19	0.59	0.30	0.29	0.06	0.19	0.01	0.01
**C18:2 c9 t11**	0.08	0.00	0.08	0.00	0.03	0.94	0.02	0.94
**C22:0**	0.36	0.05	0.29	0.06	0.06	0.66	0.01	0.51
**C20:3 n-6**	0.42	0.09	0.77	0.32	0.04	0.01	< 0.01	0.09
**C20:3 n-3**	14.38	6.21	7.48	4.29	0.52	< 0.01	< 0.01	0.01
**C20:4**	0.06	0.00	0.69	0.05	0.34	0.34	0.43	0.33
**C22:1**	0.16	0.00	0.03	0.00	0.01	0.01	0.01	0.01
**C20:5**	0.21	0.45	0.91	0.54	0.41	0.60	0.51	0.01
**C24:0**	0.15	0.15	0.21	0.06	0.11	0.93	0.17	0.01
**C22:5**	1.38	0.68	3.41	1.77	0.16	< 0.01	< .01	0.01
**C22:6**	4.93	4.93	7.75	7.35	0.30	< 0.01	0.46	0.45
**Total MUFA**	18.07	16.28	17.29	15.43	0.87	0.44	0.02	0.96
**Total PUFA**	22.97	13.45	22.18	15.73	0.82	0.40	<0.01	0.08
**Total n-3**	21.31	13.08	19.96	14.36	0.77	0.95	< .01	0.14
**Total n-6**	1.57	0.36	2.13	1.36	0.41	0.10	0.03	0.58
**Total EPA and DHA**	5.34	5.59	8.75	7.81	0.31	< 0.01	0.34	0.11
**C18:0 Desaturase**	0.43	0.50	0.46	0.46	0.02	0.73	0.15	0.16
**Ratio n-6/n-3**	0.07	0.02	0.10	0.10	0.03	0.13	0.45	0.53
**Total**	2.42	13.95	2.08	14.53	0.99	0.90	< 0.01	0.65

^a^MUFA = EnerGII as a source of palmitic and oleic acid (Virtus Nutrition LLC, Corcoran, CA): EPA+DHA = StrataG113 as a source of EPA and DHA (Virtus Nutrition LLC, Corcoran, CA).

^†^n-3 = omega-3; n-6 = omega-6; EPA = eicosapentaenoic acid; DHA = docosahexaenoic acid.

There was a treatment effect (*P <* 0.05) on C18:1 t6,8, C18:1 t12, and C20:3 n-3. The concentration of these FA in the FL and FCNS of MUFA-supplemented ewes was greater than that in the FL of EPA+DHA-supplemented ewes. There was a greater (*P <* 0.05) concentration of C20:3 n-6, C22:5, C22:6, and total EPA and DHA in the FL and FCNS of EPA+DHA- supplemented ewes compared with the FA concentration of fetuses from MUFA-supplemented ewes (Table 4). There was also a tendency for an increase (*P <* 0.10) of C16:0 *iso* concentration in the FL and FCNS due to MUFA supplementation of the dam diets. The concentration of C18:2c9t12 and total n-6 tended to increase (P < 0.10) when dams were fed the EPA+DHA diet compared with fetuses in which dam diets were supplements with MUFA (Table 4).

### Relative abundance of mRNA in the FL as a result of fatty acid supplementation

The relative abundance of LPL mRNA transcript in the FL was greater (*P =* 0.02) in the FL of dams fed diets with MUFA supplementation compared with ewes fed the EPA+DHA- supplemented diet (Table 5). The relative abundance of *FATP-1* tended (*P =* 0.10) to increase in the FL of fetuses of the dams fed MUFA-supplemented diets compared with fetuses from dams fed the EPA+DHA supplemented diet (Table 5).

**Table 5:** Relative abundances of mRNA transcripts in the fetal liver tissue of fetus from ewes fed Ca salts of a palmitic fatty acid distillate (MUFA) (n = 6) or Ca Salts of containing EPA and DHA (EPA+DHA) for 45 days (n = 6).

Treatment ^a^				
Item ^b^	MUFA	EPA+DHA	SEM	P-Value
*DGAT1*	359.06	446.58	44.78	0.30
*DGAT2*	124.99	92.36	13.23	0.22
*DNMT1*	1327.82	1243.84	73.69	0.50
*DNMT2*	239.34	284.05	29.45	0.30
*DNMT3a*	277.24	287.75	30.52	0.83
*DNMT3b*	127.29	112.89	11.49	0.46
*ELOVL2*	339.75	358.93	58.26	0.82
*ELOVL5*	3299.26	2997.57	219.42	0.35
*FABP-1*	26442	28017	3931.34	0.80
*FABP-4*	2.07	2.52	0.64	0.63
*FABP-5*	1662.22	1541.85	221.78	0.73
*FADS-1*	2097.58	2109.14	200.87	0.97
*FAT/CD36*	718.90	787.00	51.47	0.44
*FATP-1*	29.92	45.22	3.76	0.10
*FATP-4*	136.51	145.56	9.75	0.52
*FFAR-1*	19.57	22.73	4.43	0.66
*FFAR-4*	11.42	10.09	4.36	0.84
*GLUT1*	14235	14161	1370.62	0.97
*IGF-1*	37.01	37.69	6.77	0.94
*IGF-2*	57602	61691	5004.48	0.62
*LIPG*	179.98	166.11	29.74	0.77
*LPL*	103.74	79.21	6.40	0.02
*Leptin*	4.09	5.62	2.53	0.67
*PPAR β*	40.07	36.51	4.42	0.62
*PPAR α*	2223.34	1978.62	130.86	0.21
*PPAR γ*	151.42	160.99	32.36	0.85
*SCD*	1910.32	1734.94	129.90	0.36
*SREBP-1*	960.75	1011.51	52.69	0.51

^a^ MUFA = EnerGII as a source of palmitic and oleic acids (Virtus Nutrition LLC, Corcoran, CA); EPA+DHA = StrataG113 as a source of EPA and DHA (Virtus Nutrition LLC, Corcoran, CA)

^b^ DGAT1 = Diacylglycerol O-acyltransferase 1; DGAT2 = Diacylglycerol O-acyltransferase 2; DNMT1 = DNA methyltransferase 1; DNMT2 = DNA methyltransferase 2; DNMT3a = DNA methyltransferase 3a; DNMT3b = DNA methyltransferase 3b; ELOVL2 = Fatty acid elongase 2; ELOVL5 = Fatty acid elongase 5; FABP-1 = Fatty acid binding protein 1; FABP-4 = Fatty acid binding protein 4; FABP-5 = Fatty acid binding protein 5; FADS1 = Fatty acid desaturase 1; FAT/CD36 = Fatty acid translocase; FATP-1 = Fatty acid transport protein 1; FATP-4 = Fatty acid transport protein 4; FFAR-1 = Free fatty acid receptors 1; FFAR-4 = Free fatty acid receptors 4; GLUT-1 = Glucose transporter 1; IGF-1 = Insulin like growth factor 1; IGF-2 = Insulin like growth factor 2; LIPG = Endothelial lipase precursor, LPL = Lipoprotein lipase; PPAR β = Peroxisome proliferator activated betha; PPAR α = Peroxisome proliferator activated alpha; PPAR γ = Peroxisome proliferator activated gamma; SCD = Stearoyl CoA desaturase; SREBP-1 = Sterol regulatory element binding protein 1.

### Relative abundance of mRNA in the placenta after different fatty acid supplementations

There was a tissue by treatment interaction (*P =* 0.01) for the relative abundance of *FFAR-4* mRNA. For fetuses from MUFA-supplemented dams, the relative abundance of *FFAR-4* mRNA transcripts was greater in the caruncle; and for fetuses from EPA+DHA-supplemented dams, the relative abundance of *FFAR-4* mRNA transcript was greater in the cotyledon (Table 6). For *DNMT3a*, there was a tendency for a tissue and treatment interaction (*P =* 0.09). The relative abundance of DNMT3a mRNA transcript was greater in the caruncle of fetuses from MUFA-supplemented dams compared with the cotyledon of fetuses of the same treatment group and compared with the caruncle and cotyledon of fetuses from EPA+DHA-supplemented dams (Table 6). There was a treatment (*P =* 0.05) effect for the relative abundance of *FABP-4* mRNA transcript. Fetuses from MUFA-supplemented dams had a greater relative abundance of *FABP-4* mRNA in the caruncle and cotyledon than fetuses from EPA+DHA-supplemented ewes (Table 6).

**Table 6:** Relative abundance of mRNA transcripts in placenta tissues (caruncle compared with cotyledon) of lambs of ewes fed Ca salts of palmitic fatty acids distillate (MUFA) (n = 6) or Ca Salts of containing EPA and DHA (EPA+DHA) for 45 days (n = 6).

	Treatment ^a^							
Tissue	MUFA		EPA+DHA		S.E.M	*P-Values*		
Item ^b^	Caruncle	Cotyledon	Caruncle	Cotyledon		Treatment	Tissue	Tissue x Treatment
***DGAT1***	193.64	158.24	182.67	179.37	13.85	0.72	0.17	0.26
***DGAT2***	14.63	15.35	11.66	20.96	4.65	0.78	0.29	0.36
***DNMT1***	653.08	521.55	624.85	711.28	95.93	0.41	0.81	0.27
***DNMT2***	463.34	394.98	446.31	383.30	34.17	0.68	0.07	0.93
***DNMT3a***	292.08	219.31	248.25	241.27	18.76	0.57	0.04	0.10
***DNMT3b***	104.80	80.44	110.79	112.97	16.35	0.25	0.51	0.43
***ELOVL2***	146.96	345.48	133.32	744.84	267.16	0.48	0.15	0.45
***ELOVL5***	2614.52	2245.05	2606.63	2576.63	246.61	0.52	0.43	0.50
***FABP-1***	18.34	39.21	15.40	20.44	17.60	0.54	0.47	0.66
***FABP-4***	80.25	90.30	52.12	57.50	11.83	0.05	0.74	0.28
***FABP-5***	58825.0	43066.0	56891.0	28980.0	6887.1	0.26	0.01	0.39
***FADS1***	1015.28	785.61	907.22	808.71	109.47	0.73	0.09	0.49
***FAT/CD36***	11293.0	8401.1	11909.0	6807.1	1641.2	0.77	0.02	0.51
***FATP-1***	92.01	69.77	87.68	71.15	9.40	0.88	0.05	0.76
***FATP-4***	115.94	100.50	116.84	127.62	12.78	0.29	0.86	0.32
***FFAR-1***	11.38	7.98	4.90	9.34	4.97	0.64	0.91	0.39
***FFAR-4***	27.63	21.40	15.90	38.78	5.07	0.58	0.12	0.01
***GLUT1***	12582.0	7673.55	12545.0	6661.29	1428.45	0.72	< 0.01	0.74
***LPL***	110.18	121.38	97.96	122.26	24.84	0.82	0.48	0.79
***Leptin***	13.41	9.42	7.14	12.63	5.72	0.79	0.90	0.42
***PPAR* β**	80.91	66.59	73.02	73.59	7.84	0.96	0.39	0.35
***PPAR* α**	559.97	439.18	522.89	428.73	43.64	0.64	0.01	0.70
***PPAR* γ**	4824.36	2926.17	4368.79	1825.85	865.25	0.38	0.02	0.71
***SCD***	2095.82	1441.98	2017.76	1271.14	192.33	0.53	< 0.01	0.81
***SREBP-1***	1729.03	1137.12	1630.81	1166.53	103.34	0.74	< 0.01	0.54

^a^ MUFA = EnerGII as a source of palmitic and oleic acids (Virtus Nutrition LLC, Corcoran, CA); EPA+DHA *= StrataG113 as a source of EPA and DHA (Virtus Nutrition LLC*, *Corcoran*, *CA)*

^b^ DGAT1 = Diacylglycerol O-acyltransferase 1; DGAT2 = Diacylglycerol O-acyltransferase 2; DNMT1 = DNA methyltransferase 1; DNMT2 = DNA methyltransferase 2; DNMT3a = DNA methyltransferase 3a; DNMT3b = DNA methyltransferase 3b; ELOVL2 = Fatty acid elongase 2; ELOVL5 = Fatty acid elongase 5; FABP-1 = Fatty acid binding protein 1; FABP-4 = Fatty acid binding protein 4; FABP-5 = Fatty acid binding protein 5; FADS1 = Fatty acid desaturase 1; FAT/CD36 = Fatty acid translocase; FATP-1 = Fatty acid transport protein 1; FATP-4 = Fatty acid transport protein 4; FFAR-1 = Free fatty acid receptors 1; FFAR-4 = Free fatty acid receptors 4; GLUT-1 = Glucose transporter 1; IGF-1 = Insulin like growth factor 1; IGF-2 = Insulin like growth factor 2; LPL = Lipoprotein lipase; PPAR β = Peroxisome proliferator activated betha; PPAR α = Peroxisome proliferator activated alpha; PPAR γ = Peroxisome proliferator activated gamma; SCD = Stearoyl CoA desaturase; SREBP-1 = Sterol regulatory element binding protein 1.

## Discussion

The first aim of the current study was to determine if there was an effect of supplementation with Ca Salts of MUFA or EPA+DHA during the first 45 days of gestation in ewes on the patterns of fatty acids in the FL and central nervous tissues. The brain begins developing in an early stage of pregnancy, and it continues to develop throughout the postnatal period. Polyunsaturated fatty acids are important compounds in cell membranes of the central nervous tissues, therefore in brain development [14]. Docosahexaenoic acid regulates membrane permeability and improves photoreceptor differentiation during the last trimester of the pregnancy [15]. Children from mothers who consumed a small amount of fish oil had greater cognitive, visual, and behavioral problem risks [24]. The hypothesis for the present study, therefore, was that increasing EPA and DHA in the dam diet would increase the concentration of the PUFA in fetuses compared with the fetuses from MUFA-supplemented ewes. Furthermore, it was hypothesized that the total PUFA concentration would be greater in the brain than liver; and in both tissues greater than in the liver and brain of fetuses from ewes supplemented with a MUFA source of FA. Results of the present study indicate PUFA concentration was greater in the liver than in the brain of the fetus. When EPA and DHA were supplemented to ewes during the first 45 days of gestation, the PUFA concentration in the entire fetal brain was greater compared with the brain of the fetus from ewes supplemented with MUFA. Similarly, the concentrations of EPA, docosapentaenoic acid, and tetracosanoic acid were greater in the liver of fetuses from EPA- and DHA-supplemented ewes. Even though the essential fatty acids are important for brain development, there is not a physiological explanation for the greater content of the fatty acids in the liver compared to the brain. It is possible that there is a regulation of uptake and storage of FA in the tissues. This could be regulated by enzyme activity. Some of the differences could be attributed to activity of Δ5 and Δ6 desaturases in FL from early in gestation, but the activity of these enzymes appears to be less before birth than earlier in the gestation period [25]. Consumption of small amounts of PUFA during gestation results in lesser amounts of 22:6n-3 in the fetal brain and liver, as well as in the placenta of rats [26]. The mechanism that regulates the uptake of FA in the different fetal tissues and the association with maternal diet, therefore, needs further investigation.

Supplementing the maternal diet of ewes during the first 45 days of gestation with EPA and DHA tended to result in a greater abundance of *FATP-1* mRNA transcript in the FL. The presence of *FATP-1* was reported by Desantadina et al. [27] during the initial two thirds of the gestation period in the caruncle area of the placenta of cattle and this finding is consistent with those of the present study. Feeding of diets containing greater amounts of fat lead to greater abundances of placental *FATP-1* mRNA transcript compared with what is present in the placenta of animals fed standard diets [28], which may lead to increased transport of dietary fatty acids from the dam to the fetus. The changes indicate *FATP-1* might have an important function in FA metabolism in early gestation. When there was an *FATP-1*-knockout procedure imposed in mature animals, there was an increase in liver weight and triglyceride content, compared with wild type mice [29]. In the present study, there was not any association detected between total FA concentration and FATP-1 mRNA transcript abundance, however, there was not evaluation of the total triglyceride of the FL, and in the previous study there was not evaluation of the pattern of the FA in the liver tissue [29].

Supplementation with MUFA resulted in a greater abundance of LPL mRNA transcript in the FL. There are similar results in adult animals [30]. It is possible that the lesser concentration in PUFA in the liver of fetuses of MUFA-supplemented ewes is due to a greater uptake of other FA induced by LPL. In humans, LPL is a candidate gene for obesity, based on the function of the protein encoded by this gene to induce absorption of fatty acids across the cell walls of tissues. When there was supplementation of conjugated linoleic acid, there was confirmation of the capacity of this compound to reduce LPL activity, indicating that the inhibition of LPL activity seems to be a mechanism underlying body fat reduction [31].

In the present study, the expectation was that the concentration of FA in liver would be associated with abundances of mRNA transcripts for genes associated with metabolism and transport; however, the results from the current study indicate there is no association between liver FA concentration patterns and abundances of mRNA transcripts for these genes. It is possible the activity of these genes is not associated with the amount of mRNA, or that the concentration of liver FA is regulated by other factors that were outside of the scope of the variables evaluated in the study for which results are being reported in this manuscript.

The placenta functions as an interface between the dam and fetus and function in the exchange of nutrients between the dam and fetus, thus maintaining a placental microenvironment [5]. Some fetal programing effects, such as changes in DNA methylation are due to the interaction of placenta metabolism and maternal nutrient intake [32]. Results of the present study indicate there is a tendency for *DNMT3a* treatment x tissue mRNA transcript abundance because the mRNA for *DNMT3a* was greater in the caruncle of ewes supplemented with MUFA. Dekker et al. [33] reported that there was a greater placental *DNMT3a* transcript abundance for genes involved in fatty acid metabolism when there was supplementation fat in the diet. There are some inconsistences in the results regarding the effect of dietary fat and abundance of *DNMT3a* transcript. Results from other studies [10, 11] indicate there is no decrease in the relative abundance of *DNMT3a* transcript when there is feeding of a diet supplemented with fat. In the present study, there was not an assessment of DNA methylation. It is possible that the difference in results among studies is not due to the amount of FA in the diet, but more specific to the type of FA. Changes in DNA methylation are associated with changes in gene expression [10]. In the present experiment, there was no association between the abundance of mRNA *DNMT3a* and abundance of mRNA transcript for other genes. Nevertheless, the molecular mechanisms for MUFA mediated changes in *DNMT3a* gene expression in the caruncle is not well understood.

To the best of our knowledge, the results reported from the current study are the first reported for FFAR-4 in the ewe placenta. In only three published papers, has there been reporting of *FFAR-4* gene transcript abundances and these have been for tissues of cattle and mice [8, 9, 34]. The relative abundance of *FFAR-4* mRNA transcript was the greatest in the cotyledon of EPA- and DHA-supplemented ewes, followed by the caruncle of MUFA-supplemented ewes. The relative abundance of FFAR-4 mRNA is associated with obesity in human adipose tissues [8, 9], which might be increased as a result of the oxidation of lipids [9]. Animals with a larger lipid intake had a lesser abundance of placental *FFAR-4* mRNA transcript during gestation only when there was a male fetus [35]. The mechanisms that regulate the amount of FFAR-4 seems to be multifactorial, therefore, more studies need to be conducted to evaluate these mechanisms.

The relative abundance of *FABP-4* mRNA transcript was greater in the caruncle and cotyledon on the MUFA-supplemented ewes in the present study. This result is not consistent with expectations based on studies in humans [36, 37]. The protein encoded for by the *FABP-4* gene has a great amount of affinity for DHA and PUFA and it is an exclusive mechanism transporting fatty acids into placenta tissues [37]. Larqué et al. [5] reported that *FABP-4* is consistently functions in the transport of fatty acids through the placental tissues to the fetus, however, only in the transport of DHA and arachidonic acid. There, however, is no specific information about the relationship between monounsaturated fatty acids and the regulation of the *FABP-4* gene in the placenta. Based on results from the present study, it is suggested that there be further studies focused on tissue functionality and the potential functions of the protein encoded for by the *FABP-4* gene on placenta fatty acid transport when different fatty acids are supplemented during early gestation.

## Conclusion

Supplementation with an enriched source of EPA+DHA during early gestation increased the concentration of the total EPA and DHA on FL and the brain. These effects were not observed in changes of mRNA transcript abundance of genes involved in transport and metabolism of lipids in FL with supplementation with an enriched source of EPA + DHA. The only increase of the relative abundance of mRNA transcript was that for the *LPL* gene with an enriched source of MUFA compared with EPA+DHA in the FL. Fatty acid concentration in the liver and brain was not associated with changes in expression of some of the liver and placenta genes. Supplementation with an enriched source of MUFA altered the relative abundance of *DNMT3a* mRNA transcript compared with EPA+DHA supplementation. Furthermore, supplementation with an enriched source of MUFA resulted in a greater relative abundance of *FABP-4* and *FFAR-4* transcript abundances in the two sections of the placenta (caruncle and cotyledon). As far as we are aware, the present study in the first in which there is reporting of how MUFA supplementation changes the relative abundance of *FABP-4* mRNA transcript in the placenta; and in which there is report of the presence of *FFAR-4* in the placenta. Mechanisms by which supplementation of MUFA may have increased the expression of lipid transport genes requires further investigation.

## Supporting information

S1 Data (XLSX)
